# Correction to “Inflammation Is a Key Risk Factor for Persistent Seizures in Neurocysticercosis”

**DOI:** 10.1002/acn3.70245

**Published:** 2026-01-20

J. A. Herrick , B. Maharathi , J. S. Kim , et al., “Cysticercosis Working Group in Peru. Inflammation is a key risk factor for persistent seizures in neurocysticercosis,” Annals of Clinical and Translational Neurology 5, no. 5 (2018): 630–639, 10.1002/acn3.562.29761125 PMC5945963

In the author list, the name “Gerardo G. Abundis” was incorrect. Abbreviation as consequence is also wrong “Abundis GG.” The correct name should be “Gerardo F. Gomez‐Abundis.” Just to ensure clarification, my first name is Gerardo, middle initial F, and last name Gomez‐Abundis with corresponding abbreviation “Gomez‐Abundis GF.”

We apologize for this error.

